# Differential metabolomics analysis allows characterization of diversity of metabolite networks between males and females

**DOI:** 10.1371/journal.pone.0207775

**Published:** 2018-11-30

**Authors:** Zimin Li, Yuxi Zhang, Ting Hu, Sergei Likhodii, Guang Sun, Guangju Zhai, Zhaozhi Fan, Chunji Xuan, Weidong Zhang

**Affiliations:** 1 School of Pharmaceutical Sciences, Jilin University, Changchun, China; 2 Department of pharmacy, Daqing people's hospital, Daqing, China; 3 Department of pharmacy, Daqing oil-field general hospital, Daqing, China; 4 Department of Computer Science, Memorial University, St John’s, NL, Canada; 5 Provincial Toxicology Centre, Provincial Health Services Authority, Vancouver, British Columbia, Canada; 6 Discipline of Medicine, Faculty of Medicine, Memorial University, St. John’s, NL, Canada; 7 Discipline of Genetics, Faculty of Medicine, Memorial University, St. John’s, NL, Canada; 8 Department of Mathematics and Statistics, Memorial University, St. John’s, NL, Canada; 9 Northeast Asian Studies College, Jilin University, Changchun, China; University of Pittsburgh Graduate School of Public Health, UNITED STATES

## Abstract

Females and males are known to have different abilities to cope with stress and disease. This study was designed to investigate the effect of sex on properties of a complex interlinked network constructed of central biochemical metabolites. The study involved the blood collection and analysis of a large set of blood metabolic markers from a total of 236 healthy participants, which included 140 females and 96 males. Metabolic profiling yielded concentrations of 168 metabolites for each subject. A differential correlation network analysis approach was developed for this study that allowed detection and characterization of interconnection differences in metabolites in males and females. Through topological analysis of the differential network that depicted metabolite differences in the sexes, we identified metabolites with high centralities in this network. These key metabolites were identified as 10 phosphatidylcholines (PCaaC34:4, PCaaC36:6, PCaaC34:3, PCaaC42:2, PCaeC38:1, PCaeC38:2, PCaaC40:1, PCaeC34:1, PC aa C32:1 and PC aa C40:6) and 4 acylcarnitines (C3-OH, C7-DC, C3 and C0). Identification of these metabolites may help further studies of sex-specific differences in the metabolome that may underlie different responses to stress and disease in males and females.

## Introduction

It is well known that women and men react in different ways to stress, show distinctive clinical presentations in a number of conditions, and generally have dissimilar abilities of coping with illnesses [1,2]. Coronary heart disease, for example, has a higher prevalence in men than in women; this difference is even more striking if only pre-menopausal women and age-matched men are included in the comparison [3]. The latter observation contrasts with findings that the risk of developing stroke and heart failure is higher in women than in men [4,5]. The occurrence and severity of knee osteoarthritis (OA) also appears to be influenced by sex, with older females affected to a greater degree by the disease compared to age-matched males [6,7]. In our own investigations, we found that a large number of key biochemical metabolites studied in subjects affected by OA show gender-specific associations [8,9]. Considering that the development of heart diseases, obesity, and OA and other conditions is accompanied by changes in the biochemical makeup of organs and tissues, metabolic disturbances can be either the result of pathological changes or a factor contributing to the pathogenesis, or both. Sex, in combination with multiple other factors such as genetics and environmental influences may play differential effects, including those via influences on biochemical metabolism, in the onset, development and outcomes of a disease.

As a method of analysis, metabolomics involves comprehensive studies of large sets of biochemical metabolites in various states and conditions that allow investigations of complex mechanisms underlying biological functions and phenotypes [9,10]. In the analysis of composite metabolomics data, complex metabolite-phenotype relationships are often revealed by using either principal component analysis (PCA), partial least square discriminant analysis (PLS), or other techniques that allow binary class discriminations [11]. However, to understand the roles of metabolites in complex human physiological status, just as genes, they need to be studied in the context of the regulatory systems they are involved in [12]. These regulatory networks can provide the cellular context of all interested metabolites and give a means to identify specific subnetworks that are dysfunctional in a given disease or physiological state. However, those are infrequently used, largely because of limited availability of accepted methodologies [13]. For example, metabolic analysis of correlations between concentrations of metabolites has not seen wide adoption due to the lack of accepted methods of analysis. Investigations of such correlations, however, would be of great interest and may provide information about intricate interconnections between the components of complex biochemical systems.

With the goal of elucidating the complex relationships between components of metabolic makeup that are explicitly associated with sex, our specific objective in this work was further development of a method for characterizing the interconnections of metabolite pairs exhibiting significant differences in males and females. We term this method as the differential correlation network approach. Our methodology is sufficiently distinct from other existing types of approaches to analysis of metabolomics data. Through the topological analysis of differential associations of metabolite concentrations in males and females, we were able to identify the key metabolites that appear to play central roles in controlling network functional connectivity and information flow. Identification of these significant metabolites and associations should help our understanding of the foundation for sex-related effects in health and disease.

## Patients and methods

### Subjects

The subjects for this study were recruited from the cohort of Newfoundland, Canada [14]. All of the participants were adult healthy volunteers. Inclusion criteria are: 1) at least a third generation Newfoundlander, 2) not pregnant at the time of study. These people’s medical information was collected by a self-administered questionnaire. This study was approved by the Health Research Ethics Authority of Newfoundland and Labrador.

### Demographics and anthropometrics

The demographic and medical information of the study subjects was collected using a self-administered questionnaire with the help of the research staff when necessary. Anthropometric data including height and body weight were retrieved using hospital admission and/or medical records where applicable. The body mass index (BMI) was calculated by dividing weight in kilograms by squared height in meters. Age of each participating subject was recorded at the time of the blood sample collection.

### Plasma sample preparation

The EDTA-containing vacutainer tubes were used for sample collection. Blood was collected after an overnight (minimum 8 hours) fast. Plasma was separated from red cells immediately after collection by centrifugation using a standard protocol. The centrifugation was performed at 20,000 rpm for 10 mins and the plasma was immediately transferred into a clean polypropylene tube in which it was stored capped at -80°C until analysis.

### Metabolomics data collection

Metabolic profiling was performed on plasma samples in the batch mode by using the Waters XEVO TQ MS mass spectrometry system (Waters Limited, Mississauga, Ontario, Canada). Samples together with the Quality Control material at three concentration levels and calibrators were extracted, internal standard added and data question files exported for data processing. The analysis and data processing were performed with the help of the commercial reagent kit Biocrates AbsoluteIDQ p180. The kit allows quantitative analysis of a metabolic panel consisting of 186 metabolites; these included 90 species of glycerophospholipids, 40 species acylcarnitines (including free L-carnitine), 21 species of amino acids, 19 species biogenic amines, 15 species of sphingolipids and one hexose of which 90% was glucose. Additional details on the reagent kit, data acquisition and processing were previously described [15].

### Statistical methods

#### Differential analysis of metabolite correlations

Before applying the differential correlation analysis, certain pre-processing steps were conducted on the metabolomic data. First, missing entries in the data were imputed using the mean values separately for different sex. Then, we performed covariant adjustment on BMI and age, in order to remove their confounding effects. Lastly, metabolite concentration values in the population were normalized to zero mean and unit standard deviation. Next, we analyzed correlations between pairs of metabolites in females and males using Pearson’s correlation coefficient *r*. The analysis yielded correlation coefficients *r*_female_ and *r*_male_ for the two sex groups. These coefficients were subsequently used to compute changes in the correlation strength between the paired metabolites across two different sex categories. Specifically, for any two metabolites *i* and *j*, the differential correlation *r*_diff_ (*i*, *j*) was calculated as normalized difference of the Fisher’s z-transformations of *r*_female_ (*i*, *j*) and r_male_ (*i*, *j*) [16–18].

$$rdiff(i,j)=\sqrt{\frac{n_{female-3}}{2}}\times Z_{female}(i,j)-\sqrt{\frac{n_{male-3}}{2}}\times Z_{male}(i,j)$$ (1)

Where z is the Fisher’s z-transformation of correlation coefficient r,

$$Zfemale(i,j)=\frac{1}{2}ln[\frac{1+r_{female}(i,j)}{1-r_{female}(i,j)}],Zmale(i,j)=\frac{1}{2}ln[\frac{1+r_{male}(i,j)}{1-r_{male}(i,j)}]$$ (2)

We used *n*_female_ and *n*_male_ to denote the total number of samples in the female and male groups, respectively. This approach should capture the change of the normalized correlations across the two dissimilar conditions. We applied this method to evaluate whether the paired metabolites are differentially correlated by comparing males and females. Note that *r*_diff,_ found by subtracting the correlation in male from that in female describes a change of correlations, i.e. this value can be either positive or negative. A 1000-fold permutation test was applied to evaluate the significance of each differential correlation [19]. In order to remove associations among metabolite correlations and sex status, we randomly shuffled the sex status of all samples for each permutation.

#### Differential correlation network

Only metabolite pairs showing significant differential correlations were used to construct the networks. In a network constructed this way, each node represented a metabolite while the “edges” linking metabolite pairs represented significant differential correlations between the components of the network. The differential correlations of each of the metabolite pairs calculated in this approach can take either a positive or negative value, the sign meaning that the correlation is either stronger in females than in males or vice versa. The network visualization was generated using Cytoscape.30.

#### Identification of key metabolites in the differential correlation network

In network and graph analysis, indicators of centrality, node degree, betweenness centrality and closeness centrality are the most important metrics [20]. Centrality captures the importance of a discrete node in a network while the betweenness centrality is a measure of centrality that describes the number of times the shortest path between any pair of nodes, represented by $\sum s\neq v\neq t\in V\frac{\sigma_{st}(\nu )}{\sigma_{st}}$, crosses a node *v*, where σ_st_ is the number of all shortest paths from a node *s* to node *t*, and σ_st_(v) is the number of all paths that pass through the node *v*. Closeness centrality is defined as $\frac{1}{\sum_{s\neq \nu }d_{\nu s}}$, where *d*_vs_ is the distance between nodes v and s. Betweenness captures the degree to which nodes stand between each other [21]. This metric is a measure of the degree to which a given node is near other nodes in a network. Centrality in the differential correlation networks is used to identify key metabolites with important roles in the overall interrelated structure. Highly connected nodes are the nodes with high degrees, referred to as “network hubs” to indicate that they have more connections than other nodes. Nodes with high betweenness or closeness are referred to as “bottlenecks” to indicate that they have crucial roles in controlling the information flow in the network.

## Results

### Demographics and anthropometrics

A total of 140 healthy females and 96 healthy males were included in the study, with no diseases reported by the participants. The mean age was 48.0±12.6 years in females and 51.0±12.8 years in males, and the mean BMI was 29.1±5.5 kg/m^2^ in female and 28.9±4.3 kg/m^2^ in male. There were no significant differences between males and females in terms of age and BMI.

### Metabolite correlations in male and female populations

The pairwise Pearson’s correlations for the 168 metabolites were calculated using the dataset assembled from metabolite concentrations measured in all samples included in the study. We used a p-value threshold of 0.05 to define statistical significance. Close to 80% of metabolite pairs in females were positively (or negatively) correlated pairs, and a similar observation was made in datasets constructed for males. This similarity of observed numbers of correlated pairs in females and males suggests that these matching correlations were likely related to “housekeeping” biochemical reactions unrelated to sex.

### Differentially correlated metabolites

As described in Methods, the analysis of differential correlations of all pairs of metabolites was performed by comparing correlations in females to those in males. By subtracting correlations in males from corresponding correlations in females, the sex-related differences between metabolite pairs were “magnified”, while the sex-neutral correlations cancelled out. This differential correlation approach allowed us to extract dynamic correlations that were most likely related to sex differences. As a result of this analysis, 554 pairs of metabolites were uncovered to have significant positive differential correlations while 78 pairs of metabolites showed strong and negative differential correlations (permutation testing p < 0.05) between the female and male subjects. The metabolites showing the strongest correlations with high statistical significance were PC aa C34:3 and PC aa C40:1 (rdiff = 5.73, p < 0.001), and C0 and PC aa C32:3 (rdiff = −4.53, p = 0.002).

### Differential correlation network of sex

**Fig 1:**
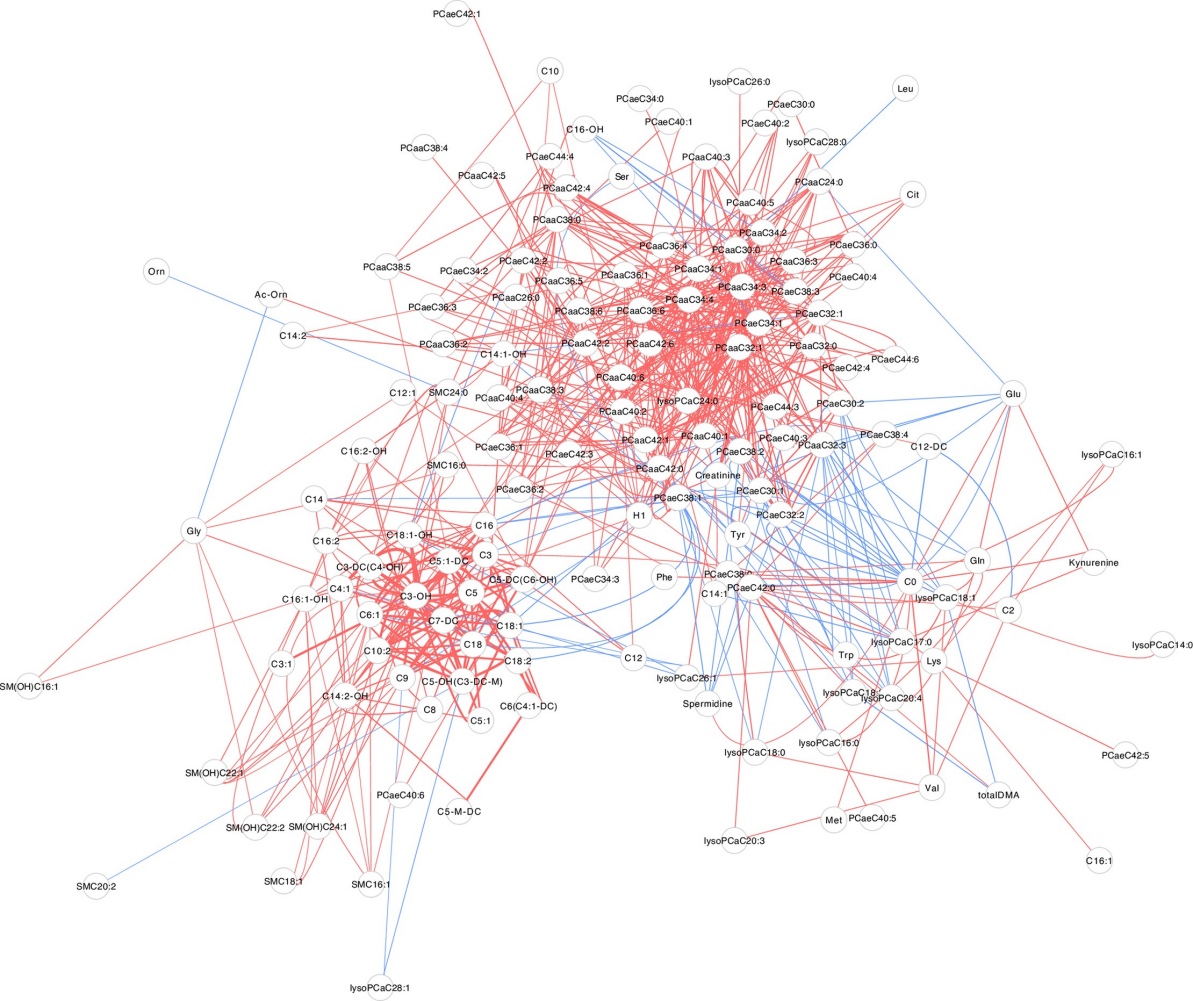
The differential correlation network showing linkages between components of the metabolite dataset. (Only pairs that have significant differential correlations are shown. The network is visualized using the force-directed layout presentation with a closer node layout distance representing a stronger pairwise correlation. Edge width is proportional to differential correlation strength and edge color shows positive (red) and negative (blue) differential correlations).

We used a set of metabolite pairs showing significant differential correlations (permutation testing significance with the cutoff of p < 0.05) to build the differential correlation network for each sex. It was found that a total of 632 pairs showed statistically significant differential correlations in this analysis, involving 146 metabolites. As seen in **Fig 1**, the majority of metabolite pairs were positively differentially correlated, as represented with red edges in the graph. Negative differential correlations were less abundant and clustered in sub-structures of the network; these links are denoted by edges of blue color in the graph. The node degree had a mean of 8.66 indicating large number of connections a typical node of this network has to other nodes suggesting robust connectivity and information flow. Overall, the graph shows two distinct clusters. The network appears to be comprised of two dense clusters and other peripheral nodes. One cluster includes mostly phosphatidylcholine metabolites and the other mostly acylcarnitine metabolites.

**Fig 2:**
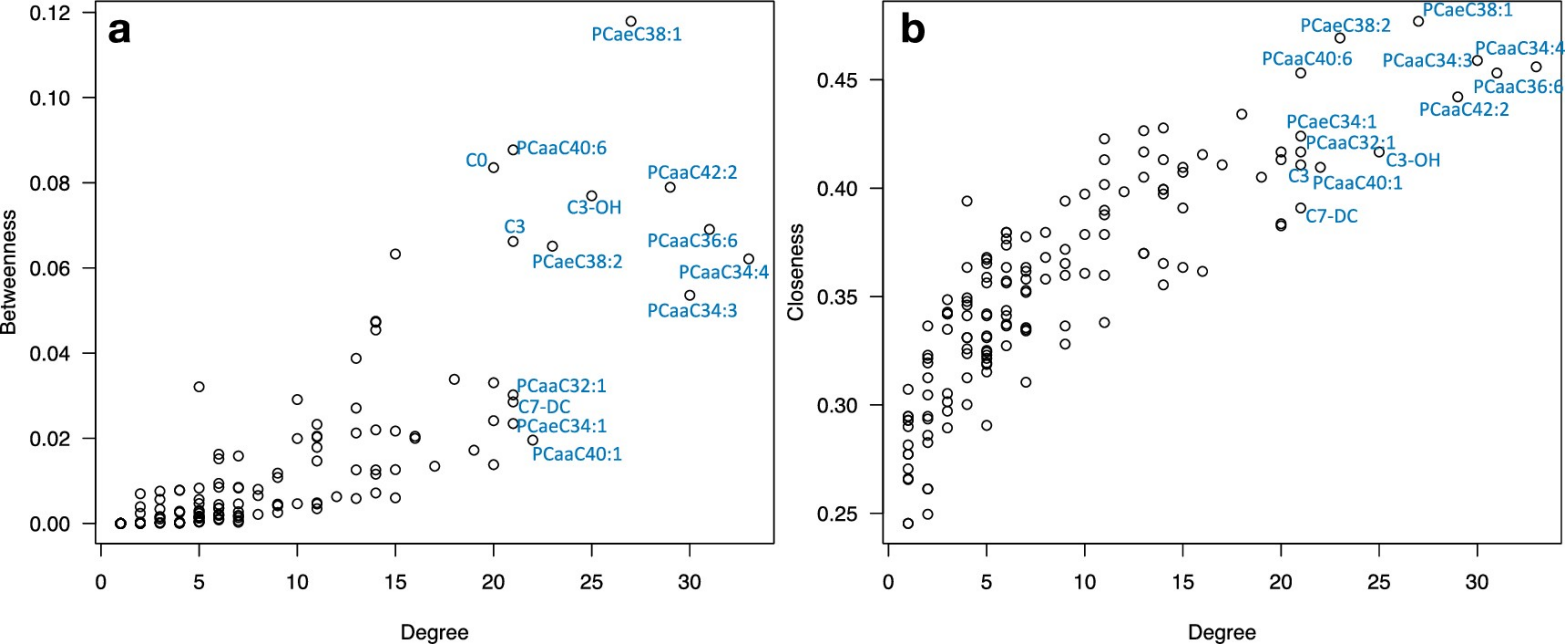
Metabolites as hubs (high degree) and bottlenecks (high betweenness or closeness) in the network.

**Fig 2** shows the *betweenness* and *closeness* centralities in relation to the node degrees. **Table 1** lists key nodes in the differential correlation network for the same set consisting of 14 metabolites. As indicated, the network model showed two main metabolite clusters. The largest cluster mainly consisted of phosphatidylcholines, of which ten metabolites were identified as key nodes (core metabolites); these were PCaaC34:4, PCaaC36:6, PCaaC34:3, PCaaC42:2, PCaeC38:1, PCaeC38:2, PCaaC40:1, PCaeC34:1, PC aa C32:1 and PC aa C40:6. The smaller cluster mainly consisted of acylcarnitines, of which C3-OH, C7-DC, C3 and C0 were identified as key nodes (core metabolites). The network diagram indicated that correlations of metabolite pairs in phosphatidylcholines’ and in acylcarnitines’ classes showed significant differences in males and females.

**Table 1:** Key nodes (metabolites) in the differential correlation network. **DC:** degree centrality; **BC:** betweenness centrality; **CC:** closeness centrality. Key nodes: either with high degrees, or high betweenness/closeness, or both.

metabolite	DC	BC	CC
PCaaC34:4	33	0.062	0.455
PCaaC36:6	31	0.069	0.453
PCaaC34:3	30	0.053	0.458
PCaaC42:2	29	0.078	0.442
PCaeC38:1	27	0.117	0.476
C3-OH	25	0.076	0.416
PCaeC38:2	23	0.065	0.469
PCaaC40:1	22	0.019	0.409
PCaeC34:1	21	0.023	0.423
C7-DC	21	0.028	0.390
PCaaC32:1	21	0.030	0.416
C3	21	0.066	0.410
PCaaC40:6	21	0.087	0.453
C0	20	0.083	0.382

## Discussion

A growing body of evidence indicates that sex is a significant factor in epidemiology, clinical presentation, and outcome in non-communicable diseases. The majority of related reports are focused on stratification of various risk factors, including sex, for such diseases as cancer, diabetes and chronic respiratory disease [22]. Other studies have looked into influence of sex differences in mounting responses of immune system to infectious diseases [23,24]. An increasing number of studies attempt to unravel specific mechanisms underlying influence of sex on disease and health outcomes [25,26]. In contrast, there are only a few studies so far that used metabolomics to investigate the influence of sex on blood serum metabolomic profiles [27–29]. This approach, however, may help to define specific risk factors such as those associated with sex and insulin resistance [27,28].

Metabolic differential correlation occurs when two metabolites show dissimilar associations between physiological/disease status. Differential correlation has been regarded as another approach to analyzing omics data, especially when individual metabolites may not show differential expression or abundance, but may be differentially associated between groups and imply a potential biological interaction [30]. Differential correlation has been previously examined in both low and high throughput studies [31,32], and has also been used with metabolomics data to identify condition-specific alterations in metabolic pathways [33,34]. Consideration of metabolites as a group of interconnected components, i.e. as a network, allows a powerful approach to the characterization of complex systems. In this study, we used network analysis to evaluate properties of the global inter-connected structure composed of metabolites that showed significant correlation variations in male and female subjects. We examined the topological properties of corresponding differential correlation networks that allowed us to identify a set of key metabolites responsible for modulating connectivity and information flow in the network. The results of this examination suggest the existence of an association between network properties and differences in the biochemical makeup of males and females.

Analysis of metabolite correlations in male and female groups of subjects showed significant overlap of correlated metabolite pairs. This observation indicated that these overlapping metabolite associations were not specifically related to sex. Further differential analysis took a unique approach that consisted in subtracting the correlation coefficients of metabolite pairs in males from those in females so that the persistent pairwise correlations across the two sex groups were removed and the pairs with significant variations were magnified. This approach was expected to provide useful new insights into the underlying sex-specific biological processes. We observed considerably larger number of significant positive differential correlations than those with negative correlations (**Fig 1**). This indicated that the number of correlations in metabolite pairs were significantly higher in females than in males.

In this study, we found phospholipids and acylcarnitines were the core metabolites in the differential network which indicated that these two species play a key role in the integrated metabolic networks of the body. Phospholipids play an essential role in the formation of a lipid bilayer in biological membranes and membrane-related phenomena such as signal transduction and regulation of membrane trafficking [35]. It has been proposed that changes in phospholipid compositions are linked to the development of OA [36]. In our previous study, we identified metabolites that are associated with OA and revealed differences of this association in females and males patients [9,37].

Acylcarnitines is a group of metabolites related to energy metabolism. Carnitine and its acyl fatty acid esters, i.e. acylcarnitines, are essential compounds for the oxidative metabolism of fatty acids. Carnitine assists in the transport of fatty acyl-CoA into the mitochondrial matrix. Deviations and abnormalities of acylcarnitine metabolism have been detected in cardiovascular diseases, type-2 diabetes and obesity [38–40]. Differences in metabolic networks in males and females that were found in this study may be one of the underlying factors that can explain differences between the sexes in their responses to stress factors and diseases.

## Conclusion

To our best knowledge, this is the first study using a differential metabolomics approach to analyze the characterization of the diversity of metabolite networks between males and females. This study shows the power of differential correlation network analysis to better understand the phenotypes of sex in the human population and to apply this knowledge in functional studies. In the study, we found that the metabolic networks of paired phosphatidylcholines and acylcarnitines do show significant differences between males and females.
